# A Biorefinery Strategy That Introduces Hydrothermal Treatment Prior to Acid Hydrolysis for Co-generation of Furfural and Cellulose Nanocrystals

**DOI:** 10.3389/fchem.2020.00323

**Published:** 2020-04-22

**Authors:** Dawit Beyene, Michael Chae, Thava Vasanthan, David C. Bressler

**Affiliations:** Biorefining Conversions and Fermentation Laboratory, Department of Agricultural, Food and Nutritional Science, University of Alberta, Edmonton, AB, Canada

**Keywords:** biorefinery, cellulose nanocrystals, furfural, xylan degradation, hydrothermal treatment, kraft pulp, crystallization

## Abstract

Hydrothermal treatment of wood pulp at 150–225°C prior to acid hydrolysis was investigated in the context of isolating cellulose nanocrystals (CNCs). The objective was 2-folds as follows: (a) generating furfural as a value–added co–product; and (b) concentrating and forming new CNC precursors through thermal re–orientation of para–crystalline cellulose chains that will in turn improve CNC recovery and yield. Furfural yields up to 19 and 21% xylan conversion were obtained at 200 and 225°C hydrothermal treatments, respectively. In addition, these hydrothermal treatment conditions increased the crystallinity index of the pulp (77%) to 84 and 80%, respectively. Consequently, the CNC yield from hydrothermally treated wood pulp, when compared to untreated wood pulp, improved by up to 4- and 2-folds, respectively. An efficient acid hydrolysis process with yield improvements can translate to reduced CNC isolation and purification costs and increased production capacity. The qualities of the CNCs in terms of particle size and crystallinity were not affected due to hydrothermal treatment. However, the zeta potential, sulfur, hydrogen, and oxygen content of the CNCs were significantly lower at 225°C while carbon composition increased, and dark brown coloration was observed that indicates caramelization. This study demonstrates for the first time a novel biorefinery strategy that introduces hydrothermal treatment prior to acid hydrolysis to co–generate furfural and CNC with improved efficiency.

## Introduction

The pulp and paper industry plays an integral role to the economy of countries that have abundant forestry resource in North America, Northern Europe, East Asia, and South America (Bajpai, 2016). The industry is currently facing multiple challenges, particularly a shrinking global market for most paper grade products (Quad, 2019). In response, the forestry sector is making efforts to generate fuels, biochemical, and biomaterial co–products from wood pulp. Cellulose nanocrystals (CNCs) are one of the value–added purified cellulosic materials that are gaining a lot of interest in research and the industry (Trache et al., 2017). Cellulose nanocrystals (CNCs) are sustainable and renewable nanoparticles derived from cellulose with desirable reinforcement, rheological, absorbant, and barrier properties for various applications. These particles represent highly ordered crystalline cellulose reduced to nanoscale length (100–200 nm) and width (5–20 nm) (Ngo et al., 2018). These materials are light–weight with mechanical strength properties comparable with carbon fiber and steel (Moon et al., 2011). Therefore, CNCs have potential application as biodegradable and renewable reinforcement fillers for composite materials used in automotive, construction and food packaging industries (Fortunati et al., 2012a; Kaboorani et al., 2012; Cao et al., 2015; Kong et al., 2016). In addition, their shear thinning, optical iridescence, high absorbance, high surface area (for grafting), oxygen and water barrier properties are also attractive for application in products used in aviation, pharmaceutical, food, cosmetics, paint, and film industries (De Souza Lima and Borsali, 2004; Shopsowitz et al., 2010; Boluk and Zhao, 2012; Chen et al., 2012; Fortunati et al., 2012b; Batmaz et al., 2014; Pereira et al., 2014; Hu et al., 2015; Jin et al., 2015; Li et al., 2016; Panchal and Mekonnen, 2019).

The cellulose structure contains crystalline, para-crystalline, and amorphous regions that have chains aligned at high, intermediate and poor level of ordering, respectively (Larsson et al., 1997). CNCs can be isolated by hydrolysis of the non–crystalline cellulose and fragmentation of crystalline cellulose to nanoscale dimensions with mineral acids, organic acids, oxidants, sub–critical water and enzymes (cellulases) (Luong et al., 2014; Filson et al., 2009; Chen et al., 2015, 2016; Novo et al., 2015; Bondancia et al., 2017). Acid hydrolysis using concentrated sulfuric acid (64 %) is the most common method for isolating CNCs in research laboratories and industrial scale facilities such as InnoTech Alberta (AB, Canada) and Melodea Ltd. (Sweden) (Chen et al., 2015; Ngo et al., 2018; Melodea Ltd., 2019). The acid degrades non–crystalline cellulose and hemicelluloses, while the highly crystalline CNC precursors are fragmented to CNCs. However, the efficiency of the acid hydrolysis process is limited due to the presence of non–crystalline cellulose and hemicellulose in the feedstock that are degraded in the acid stream. Hence the process has limitations due to low CNC yields and the loss of valuable sugars in the acid stream that also decreases the acid recovery efficiency. There is an opportunity to process the non–crystalline polysaccharide regions prior to acid hydrolysis to improve biomass utilization. Previously, we demonstrated that cellulase treatment prior to acid hydrolysis can allow recovery of sugars from preferential degradation of non–crystalline cellulose (Beyene et al., 2017). Consequently, CNC precursors were accumulated due to the preferential hydrolysis that significantly improved the CNC yield (wt % feedstock for acid hydrolysis).

Alternatively, there is a potential to introduce hydrothermal treatment prior to acid hydrolysis to generate furfural as a co–product and further improve the CNC yield. Hydrothermal treatment involves modification and partial degradation of lignocellulose biomass components (in a slurry) with water heated at 150–230°C (Garrote et al., 1999; Möller et al., 2011). Hemicellulose is primarily hydrolysed under these conditions to form sugar degradation products such as furfural and organic acids (Garrote et al., 1999; Kumar and Gupta, 2008). Furfural is a valuable platform chemical and solvent with applications in agriculture, cosmetics, and pharmaceutical industries (Mandalika and Runge, 2012; Peleteiro et al., 2016). The integration of hydrothermal treatment with acid hydrolysis to generate furfural as a co–product from degradation of hemicellulose in the feedstock is yet to be explored.

Furthermore, it has been speculated that during hydrothermal treatment, moist heat facilitates the rotation of the torsion angle (ω) of O_6_-C_6_-C_5_-O_5_ from gauche–trans (gt, ω = 60°) to trans–gauche (tg, ω = 180°) orientation in the glucose subunits (Agarwal et al., 2017). Consequently, this conformational change in structure can promote cellulose chains in para–crystalline cellulose to re–orient and crystallize. Studies have shown improvement in the crystallinity of cellulosic feedstock due to hydrothermal treatment (Isogai et al., 1991; Inagaki et al., 2010). Agarwal et al. isolated lignin containing CNC by acid hydrolysis of raw wood (composed of cellulose, hemicellulose and lignin) that was hydrothermally treated (167–225°C) (Agarwal et al., 2017). The authors reported an improvement in CNC yield and crystallinity by up to 19 and 65%, respectively. To the best of our knowledge, this is the only account in the literature of an attempt to evaluate CNC yield from a hydrothermally treated feedstock while further studies are required to exploit the hydrothermal treatment–mediated strategy. It will be interesting to explore the effect of hydrothermal treatment on a purified feedstock such as wood pulp with lower hemicelluloses and only a trace amount of lignin. Studies show that the kraft pulping process increases crystallinity of wood pulp due to: (a) partial degradation of some amorphous celluloses and hemicelluloses, and (b) an increase in reaction temperature from 105 to 160°C (Gümüskaya et al., 2003; Evans et al., 2009). Yet, it is probable that further heating through hydrothermal treatment can increase the crystallinity of cellulose beyond what could be achieved from kraft pulping process and improve CNC yield. The objectives of this study are to develop a biorefinery strategy that introduces hydrothermal treatment prior to acid hydrolysis to generate furfural as a value–added co–product and form new crystals to improve the CNC yield from Kraft pulp. The hypothesis is that hydrothermal treatment will substantially degrade xylan to furfural and also facilitates crystallization of para–crystalline chains to form new CNC precursors.

## Materials and Methods

### Feedstock and Chemicals

Northern bleached hardwood kraft (NBHK) pulp was used as a model kraft pulp feedstock. Alberta Pacific Forest Industries Inc. (Al–Pac Inc., Edmonton, AB, Canada) generously supplied dried pressed sheets, which were cut in to ~6 mm × 8 mm by a pulp chopper (Pierret Cutting Machine G45L1, Corbion, LX, Belgium) at the InnoTech Alberta (Edmonton, AB, Canada).

Acetic acid (CH_3_COOH, 99.7%) and sodium chloride (NaCl, ≥99%) were procured from Fisher Scientific (Whitby, ON, Canada). Sulfuric acid (H_2_SO_4_, 72% and 95–98%), sodium hydroxide (NaOH, 98.8%), glucose (C_6_H_12_O_6_, ≥95%), xylose (C_5_H_10_O_5_, ≥99%), furfural (C_5_H_4_O_2_, 99%), 5–methyl furfural (C_6_H_6_O_3_, 99%), formic acid (CH_2_O_2_, >95%), and calcium carbonate (CaCO_3_, >99%), were purchased from Sigma–Aldrich (St. Louis, MO, USA). Deionized water (Milli–Q® water) was used to make solutions and suspensions in all experiments.

### Hydrothermal Treatment

A Parr® High Pressure/High Temperature reactor (Parr series 4570, 1 L capacity, Parr Instrument Company, Moline, IL, USA) was used to carry out hydrothermal treatments. The reaction was controlled with SpecView® 32 Version 2.5 (SpecView Corporation, Gig Harbor, WA, USA). The reactor vessel and the reactor lines were purged with nitrogen 3 times for 5 min while maintaining a 3.4 MPa pressure in a closed system. Hydrothermal treatment of the pulp suspension in water (10 g in 400 mL, 2.5% solid loading) was carried out in batch mode at 150–225°C with a 25°C increment for 1 h with stirring (100 rpm). The starting pressure and hold time were 0.1 MPa (atmospheric pressure) and 1.5 h, respectively, in all cases. The reaction was cooled to room temperature overnight. A control group that mimics the hydrothermal treatment was also set up at room temperature while maintaining the same aqueous suspension, stirring and incubation period conditions.

### CNC Isolation

The slurry generated from hydrothermal treatment was centrifuged at 33,700 × g for 15 min at 20°C. The precipitated solid was re–suspended in 100–150 mL water and centrifuged 3 times (at the same conditions by replenishing the water) to wash off soluble degradation products. The pellet was freeze dried and the solid recovery (wt % original feedstock) was calculated using (Equation 1) in which W_1_ is the mass of pulp before hydrothermal treatment (g) and W_2_ is the mass of freeze–dried solid recovered after treatment (g).

(1) $$\begin{array}{l} \text{Solid recovery}=\frac{{\text{W}}_{2}}{{\text{W}}_{1}}\times 100\% \end{array}$$

CNC from the hydrothermally treated pulp (2.5 g, dry weight) was isolated using 64 wt % H_2_SO_4_ (31.25 mL to attain 8 % solid consistency) followed by neutralization with 30 % NaOH, dialysis in water and centrifugation to generate a purified aqueous colloid based on an InnoTech Alberta bench scale protocol that was reported in detail previously (Lorenz, 2014; Beyene et al., 2017). An aliquot (~180–200 g) of the colloid was oven dried at 105°C overnight to determine the yield based on gravimetric analysis. Equations (2)–(4) were used to calculate: a) CNC yield_1_, which was defined as the yield from a fixed mass (2.5 g) of feedstock that enters the acid hydrolysis reactor (wt % feedstock for acid hydrolysis); b) over–sized rejects (wt % original feedstock); and (c) CNC yield_2_, which was defined as the yield from the initial feedstock that undergoes treatment and acid hydrolysis whereby the mass loss due to the treatments is accounted for (wt % original feedstock), respectively, in which W_3_ is the mass of oven dried CNC from an aliquot sample (g), F_1_ is the ratio of total mass of colloid to mass of aliquot, W_4_ is the mass of the feedstock added to the acid hydrolysis reaction (g), W_5_ is the freeze–dried mass of the un–hydrolyzed pellet collected after acid hydrolysis (g), and solid recovery (wt % original feedstock) as determined in Equation (1).

(2) $$\begin{array}{l} \text{CNC yiel}{\text{d}}_{1}=\frac{{\text{W}}_{3}\;x\;{\text{F}}_{1}}{{\text{W}}_{4}}\times 100\% \end{array}$$

(3) $$\begin{array}{l} \text{Over sized reject}=\frac{{\text{W}}_{5}}{{\text{W}}_{4}}\times 100\% \end{array}$$

(4) $$\begin{array}{l} \text{CNC yiel}{\text{d}}_{2}=\frac{\text{CNC yiel}{\text{d}}_{1}\times \text{solid recovery }}{100} \end{array}$$

### Characterization Studies

#### Sugars and Other Degradation Products

The liquid recovered from hydrothermal treatments was analyzed for glucose, xylose and sugar degradation product yields using high performance liquid chromatography (HPLC, Agilent 1200, Santa Clara, CA, USA). Glucose and xylose in the sample (30 μL injection volume) were separated on an HPX−87P column (Bio–Rad Aminex, Hercules, CA, USA) with water as the mobile phase at a flow rate of 0.5 mL min^−1^ at 80°C for 40 min. The separated fractions were analyzed with a refractive index detector (RID, Agilent 1100 series, Agilent Technologies, Santa Clara, CA, USA). Sugar degradation products (furfural, hydroxymethyl furfural, acetic acid and formic acid) were separated on an HPX−87H column (Bio–Rad Aminex, Hercules, California, USA). Samples were carried through the column with 5 mmol L^−1^ H_2_SO_4_ at a flow rate of 0.5 mL/min at 60°C for 90 min. Ultraviolet and refractive index detectors were used to analyze the furans (hydroxymethyl furfural at 284 nm and furfural at 275 nm) and organic acids (acetic and formic acids), respectively (Zhang et al., 2017). Standards of the sugars (0.25–16 mg mL^−1^) and their degradation products (0.01–0.25 mg mL^−1^) were also simultaneously run on HPLC to generate a calibration curve (*R*^2^ > 0.99). Individual product yield (wt % original feedstock) was calculated using (Equation 5), in which W_6_ is the mass of product as determined from HPLC analysis (g) and W_7_ is the mass of sample used for the analysis.

(5) $$\begin{array}{l} \text{Product yield}=\frac{{\text{W}}_{6}}{{\text{W}}_{7}}\times 100\% \end{array}$$

#### Structural Polysaccharide Composition

Changes in the structural polysaccharide content in hydrothermally treated pulp were determined by a 2–step acid hydrolysis compositional analysis method (Sluiter et al., 2012). Samples (up to 2 g) were powdered on a Retsch ZM 200 Ultra Centrifugal Mill (Newton, PA, USA) operated at 8,000 rpm and passed through a 0.5 mm screen. The first step hydrolysis of pulp was carried out with 72 wt % H_2_SO_4_ (10% solid loading) at 30°C for 1 h with continuous stirring. The reaction was terminated by diluting the acid to 4% with water. Sugar recovery standard solutions were prepared to account for sugar degradation during the secondary hydrolysis. All samples were autoclaved at 121°C for 1 h at 0.1 MPa for the second step hydrolysis. After cooling the tubes, the solution was vacuum filtered on pre–weighted filtering crucibles. The filtrate was neutralized to pH 7 with CaCO_3_ powder. The suspension was centrifuged 3,300 × g for 5 min and the liquid supernatant was analyzed for sugars on HPLC (under conditions described in section Sugars and Other Degradation Products). Cellulose and xylan compositions (wt % hydrothermally treated feedstock) were determined using (Equation 6) in which W_8_ is the absolute sugar mass (mg) from HPLC analysis, F_2_ is the anhydro correction factor (0.90 for glucose and 0.88 for xylose), W_9_ is the mass of the hydrothermally treated pulp sample (mg) and R is the % recovered sugar from HPLC analysis of sugar recovery standards.

(6) $$\begin{array}{l} \text{Polysaccharide}=\frac{{\text{W}}_{8}\times {\text{F}}_{2}}{{\text{W}}_{9}\times \text{R}}\times 100\% \end{array}$$

#### Crystallinity

The degree of crystallinity of the hydrothermally treated pulp and the CNC isolated by acid hydrolysis from this feedstock were determined from X-ray diffraction analysis based on the peak height method as described previously at the nanoFAB fabrication and characterization center, University of Alberta (Beyene et al., 2018).

#### Particle Size and Colloid Stability

The hydrodynamic diameter and zeta potential of CNCs were determined from dynamic light scattering analysis of sample as discussed in our previous report at the Biomass Conversion and Processing division, InnoTech Alberta (Beyene et al., 2018).

#### Transmission Electron Microscopy Analysis

The structural characteristics of CNC particles were analyzed under transmission electron microscopy (TEM) as reported previously at the Advanced Microscopy Facility in the Department of Biological Sciences, University of Alberta (Beyene et al., 2018).

#### Elemental Composition

Freeze dried CNCs were kept in a vacuum oven at room temperature for 8 h (Abitbol et al., 2013). Carbon, hydrogen, nitrogen and sulfur compositions were determined based on the Pregl–Dumas method. The analysis was carried out on a Thermo Scientific Flash 2000 Elemental Analyzer (Themo Fisher Scientific Inc., Rodano, Milan, Italy) at the Analytical and Instrumentation Laboratory in the Department of Chemistry, University of Alberta. The oxygen content was calculated as the difference between the sum of all other elements from 100%. Approximately 1.5–1.8 mg of sample was combusted at 1,000°C in the presence of oxygen. The combustion products were carried through a gas chromatographic column (Porapak QS, 4 mm internal diameter and 2 m long) with Helium (mobile phase). Signals of the separated gases identified by a thermal conductivity detector were analyzed on Eager Xperience software (Themo Fisher Scientific Inc., Rodano, Milan, Italy).

### Data Analysis

All data were reported as mean ± standard deviation from analysis of experimental triplicates. One–way ANOVA and Tukey's test, at a 95% confidence interval (CI) was used for pairwise comparison of means on Minitab 18.1 (Minitab Inc., State College, PA, USA). An interquartile range test was used to detect any outliers.

## Results

### Degradation Profile and Crystallinity Changes Due to Hydrothermal Treatment

**Figure 1:**
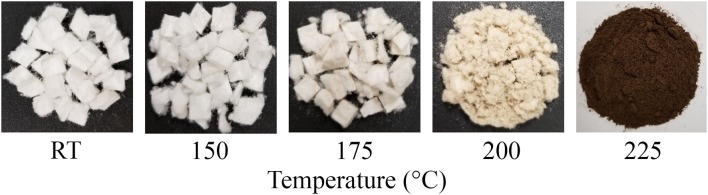
Image of control (room temperature, RT) and hydrothermally treated pulp at different temperatures (150–225°C) showing brown coloration with increasing intensity at higher temperatures (200 and 225°C), possibly due to surface binding of sugar degradation products.

To determine whether hydrothermal treatment could be successfully introduced into a biorefining platform for co–production of furfural and CNCs, square cut wood pulp was hydrothermally treated at 150–225°C for 1 h. As seen in Table 1, the pressure in the reactor vessel rose from 0.414 to 2.551 MPa, and displayed a direct relationship with temperature. Hydrothermal treatment at temperatures ≥175°C significantly decreased the solid recovery. Consequently, the square cut dimension (~6 × 8 mm) of the pulp was reduced to coarse and fine powder at 200 and 225°C, respectively (Figure 1). Furthermore, the hydrothermally treated pulps were faint yellow, light brown and dark brown colored at 175, 200, and 225°C, respectively.

**Table 1:** Reaction conditions and characteristics (solid recovery, polysaccharide composition, and crystallinity) of wood pulp that has undergone hydrothermal treatment at room temperature (control) and 150–225°C.

**Hydrothermal treatment (**°**C)**	**Recorded pressure (MPa)**	**Solid recovery (wt % original feedstock)**	**Polysaccharide composition (wt % hydrothermally treated feedstock)**		**Crystallinity index (%)**
			**Cellulose**	**Xylan**	
RT	*	98.9 ± 0.4^A^	74.3 ± 0.1^H^	19.2 ± 0.1^P^	77 ± 1^W^
150	0.414	97.3 ± 0.6^A^	75.6 ± 2.6^H^	18.8 ± 0.7^P^	78 ± 2^WX^
175	0.827	92.2 ± 0.3^B^	78.0 ± 3^HI^	15.5 ± 0.6^Q^	81 ± 2^XY^
200	1.551	78.5 ± 0.6^C^	83.6 ± 2.3^I^	7.2 ± 0.3^R^	84 ± 2^Y^
225	2.551	46.6 ± 1.2^D^	73.4 ± 0.5^H^	1.9 ± 0.2^S^	80 ± 1^WXY^

*Each data set represents the mean ± standard deviation from experimental triplicates. Values with different letters (in superscripts) within each column are significantly different (p < 0.05)*.

* *Carried out at atmospheric pressure. RT, room temperature as a control*.

Polysaccharide compositional analysis on the hydrothermally treated pulp shows that there were no changes in xylan and cellulose contents at 150°C. Very poor peak intensities of oligo–, monosaccharide, and other degradation products were detected from liquid hydrolysate analysis at this temperature (Table 2). Pulp degradation was initiated at 175°C with significant xylan hydrolysis. The xylan composition in the hydrothermally treated wood pulp (wt % hydrothermally treated feedstock) significantly decreased at 200°C (7.2 ± 0.3%) and 225°C (1.9 ± 0.2%) relative to the original feedstock (19.2 ± 0.1%) (Table 1). However, xylose recovery at 200°C (1.7 ± 0.1 wt % original feedstock) was very lower and xylose was not even detectable at 225°C.

**Table 2:** Composition of the liquid fraction generated from hydrothermal treatment of wood pulp at room temperature (control) and 150–225°C.

**Hydrothermal treatment (**°**C)**	**pH**	**Sugars and degradation product recovery (wt % original feedstock)**					
		**Glucose**	**Xylose**	**Hydroxy methyl furfural**	**Furfural**	**Formic acid**	**Acetic acid**
RT	5.0	0.0 ± 0.0	0.0 ± 0.0	0.0 ± 0.0	0.0 ± 0.0	0.0 ± 0.0	0.0 ± 0.0
150	4.5	0.0 ± 0.0	0.1 ± 0.0	0.0 ± 0.0	0.1 ± 0.0^A^	0.0 ± 0.0	0.0 ± 0.0
175	3.9	0.1 ± 0.0	0.8 ± 0.0	0.0 ± 0.0	0.3 ± 0.0^A^	0.1 ± 0.0^P^	0.0 ± 0.0
200	3.3	1.6 ± 0.1	1.7 ± 0.1	0.0 ± 0.0	3.7 ± 0.2^B^	0.2 ± 0.0^P^	0.2 ± 0.0^W^
225	2.5	7.0 ± 1.5	0.0 ± 0.0	0.0 ± 0.0	4.1 ± 0.8^B^	1.4 ± 0.2^Q^	0.4 ± 0.0^X^

*Each data set represents the mean ± standard deviation from experimental triplicates. Values with different letters (in superscripts) within each column are significantly different (p < 0.05). RT, room temperature as a control*.

Substantial concentration of furfural was generated at 200 and 225°C (3.7 ± 0.2 and 4.1 ± 0.8 wt % original feedstock) (Table 2). Formic and acetic acids were the other sugar degradation products identified in trace concentrations, while hydroxymethyl furfural was not detected. An unidentified peak, with substantial intensity was separated at 35 min retention time and detected by UV analysis at 284 and 275 nm. A reduction in the pH was observed in the liquid hydrolysate from pH 4.5 at 150°C to pH 2.5 at 225°C.

There was a significant increase in cellulose composition at 200°C relative to the control pulp. Very low glucose content (1.6 ± 0.1 wt %) and oligosaccharide peak intensities were also detected from analysis of the liquid hydrolysate. However, cellulose was significantly degraded at 225°C with glucose yields of 7.0 ± 1.5 wt % original feedstock. Interestingly, the mass balance from the 225°C hydrothermal treatment was far from complete.

Hydrothermal treatments at 175 and 200°C significantly improved the crystallinity index (81 ± 2 and 84 ± 2%, respectively) of pulp relative to the control (77 ± 1%) (Table 1). Interestingly, the crystallinity index of pulp hydrothermally treated at 225°C was not significantly different than the control.

### Cellulose Nanocrystal Yields From Hydrothermally Treated Pulp

CNC was isolated from hydrothermally treated wood pulp by acid hydrolysis. CNC yield_1_ (wt % feedstock for acid hydrolysis) improved by 1.4-fold, doubled and quadrupled due to heat treatment at 175 (13.9 ± 0.4%), 200 (19.8 ± 0.6%), and 225°C (38.3 ± 2.4%), respectively, relative to the control (9.8 ± 1.3%) (Table 3). The CNC yield_2_, which was calculated by accounting for the mass loss due to hydrothermal degradation, was significantly improved above 150°C from 9.7 ± 1.3% (control) to up to 17.8 ± 1.4% (225°C).

**Table 3:** CNC yields from acid hydrolysis of wood pulp that was hydrothermally treated at room temperature (control) and 150–225°C.

**Hydrothermal treatment(**°**C)**	**CNC yield_**1**_ (wt % feedstock for acid hydrolysis)**	**Improvement in CNC yield_**1**_(fold)**	**CNC yield_**2**_ (wt % original feedstock)**	**Improvement in CNC yield_**2**_ (fold)**
RT	9.8 ± 1.3^A^	–	9.7 ± 1.3^P^	–
150	10.1 ± 0.8^A^	1.0	9.8 ± 0.8^P^	1.0
175	13.9 ± 0.4^B^	1.4	12.8 ± 0.4^Q^	1.3
200	19.8 ± 0.6^C^	2.0	15.5 ± 0.6^R^	1.6
225	38.3 ± 2.4^D^	3.9	17.8 ± 1.4^R^	1.8

*Each data set represents the mean ± standard deviation from experimental triplicates. Values with different letters (in superscripts) within each column are significantly different (p < 0.05). RT, room temperature as a control*.

### Characteristics of Cellulose Nanocrystals From Hydrothermally Treated Pulp

**Figure 2:**
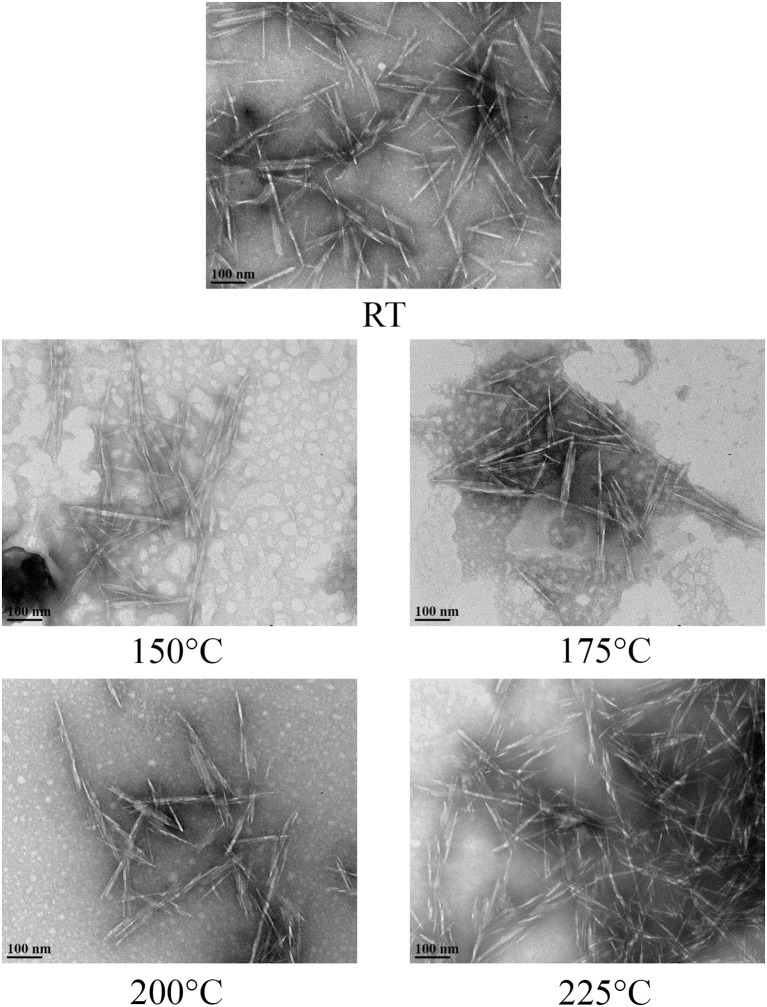
TEM micrographs of CNCs isolated from acid hydrolysis of pulp that was hydrothermally treated at room temperature (RT) as control and 150–225°C exhibited nanoscale dimension and appeared to have similar needle-like structures.

**Figure 3:**
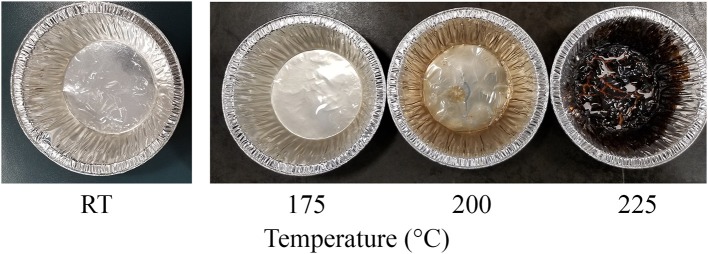
Image of CNC after oven drying colloid solution, which has been purified following acid hydrolysis of wood pulp that was hydrothermally treated at room temperature (RT) as control and 175–225°C appear brown with increasing intensity of the stain at 200 and 225°C.

The degree of crystallinity of CNC was not affected due to hydrothermal treatment when compared with the control (Table 4). The hydrodynamic diameter of CNCs from hydrothermally treated pulp ranged from 199 ± 75 nm to 320 ± 42 nm, which also showed no significant difference relative to the control (Table 4). The nanoscale dimensions were also confirmed from observations of TEM micrographs, which reveal that the CNC particles had lengths of few hundred units with widths ranging from ones to tens units (Figure 2). In addition, the particles appeared needle shaped with no visible differences between CNCs isolated from the control and hydrothermally treated pulp. The zeta potential values also did not significantly change due to treatment at 150–200°C with very close values ranging from −41.8 ± 0.8 mV to −41.7 ± 1.9 mV. However, there was significant increase in magnitude at 225°C. Elemental analysis also revealed that the CNC isolated from pulp hydrothermally treated at 225°C had lower sulfur content (0.9 ± 0.1 wt %) compared with the control (1.3 ± 0.1 wt %) (Table 5). Significant differences were also identified in other components with higher carbon content (44.2 ± 1.5 wt %) and lower hydrogen and oxygen compositions (5.56 ± 0.05 and 49.3 ± 1.4 wt %) relative to the untreated CNC (40.4 ± 0.4, 5.75 ± 0.05, and 52.5 ± 0.4 wt %, respectively). Interestingly, intense dark coloration was also observed on the CNCs from the treatment at this temperature (Figure 3).

**Table 4:** Crystallinity, particle size, and colloidal stability of CNCs isolated from wood pulp that has undergone hydrothermally treatment at room temperature (control) and 150–225°C.

**Hydrothermal treatment (**°**C)**	**Crystallinity index (%)**	**Average hydrodynamic diameter (nm)******	**Zeta potential (mV)**
			
RT	80.9 ± 2.5^A^	242 ± 58^PQ^	−41.3 ± 1.0^W^
150	81.0 ± 1.2^A^	199 ± 75^Q^	−41.8 ± 0.8^W^
175	81.1 ± 2.0^A^	255 ± 54^PQ^	−41.8 ± 0.5^W^
200	74.3 ± 9.2^A^	320 ± 42^P^	−41.7 ± 1.9^W^
225	79.0 ± 1.5^A^	254 ± 75^PQ^	−38.8 ± 2.0^X^

*Each data set represents the mean ± standard deviation from experimental triplicates. Values with different letters (in superscripts) within each column are significantly different (p < 0.05). ^†^Average diameter of the peak showing the highest abundance intensity (67–79%). The conductivity, pH, and temperature of the colloid were 0.7–0.8 mS/cm, 4.6–4.8, and 25°C, respectively. The polydispersity indices ranged from 0.6 to 0.8, which measures the broadness of the size distribution. Colloid with an index value below 0.05 is monodisperse, below 0.08 is nearly monodisperse, 0.08–0.7 is midrange value, and above 0.7 is a broad distribution (Shaw, Shaw). Data represent mean ± standard deviation of analytical triplicates of treatment triplicates. RT, room temperature as a control*.

**Table 5:** Elemental composition analysis of CNCs isolated from wood pulp that was hydrothermally treated at room temperature (control) and 150–225°C.

**Hydrothermal treatment (**°**C)**	**Elemental analysis (wt % CNC)**				
	**Carbon**	**Hydrogen**	**Nitrogen**	**Sulfur**	**Oxygen**
RT	40.4 ± 0.4^A^	5.75 ± 0.05^H^	0	1.3 ± 0.1^P^	52.5 ± 0.4^W^
150	40.5 ± 0.3^A^	5.76 ± 0.03^H^	0	1.2 ± 0.1^P^	52.5 ± 0.3^W^
175	40.4 ± 0.1^A^	5.77 ± 0.02^H^	0	1.3 ± 0.1^P^	52.5 ± 0.1^W^
200	41.1 ± 0.2^A^	5.82 ± 0.03^H^	0	1.2 ± 0.1^P^	51.9 ± 0.2^W^
225	44.2 ± 1.5^B^	5.56 ± 0.05^I^	0	0.9 ± 0.1^Q^	49.3 ± 1.4^X^

*Each data set represents the mean ± standard deviation from experimental triplicates. Values with different letters (in superscripts) within each column are significantly different (p < 0.05). RT, room temperature as a control*.

## Discussion

The water was nearly subcritical under the hydrothermal treatment conditions in the present study. The pressure values (0.414 and 0.827 MPa) generated at 150 and 175°C were below the saturation vapor pressure (0.478 and 0.894 MPa, respectively) for water to be subcritical at these temperatures (Table 1; Cengel and Boles, 2004). However, the pressures at 200 and 225°C (1.551 and 2.551 MPa, respectively) were very close to the saturation vapor pressures (1.553 and 2.548 MPa) and hence, the heated water was on the borderline with the subcritical region.

Significant degradation of pulp due to hydrothermal treatment was mainly attributed to xylan degradation (Table 1). Hemicelluloses generally solubilize at 180°C and are relatively less stable because of accessibility of glycosidic bonds with lower levels of crystallinity than celluloses (Bobleter, 1994). Bizikis et al. also reported significant loss in hemicellulose from 25 to 28% down to 4, 9, and 12% due to hydrothermal treatment of birch wood, aspen and alder wood, respectively, for 1 h at 180°C (Biziks et al., 2010). The formation of an acidic medium, evident from the decrease in pH presumably due to organic acids released from xylose degradation, has likely facilitated autohydrolysis reactions (Bobleter, 1994; Boonstra et al., 2007; Kumar and Gupta, 2008; Huerta and Saldaña, 2018). With significant xylan degradation, cellulose accumulation was apparent in the hydrothermally treated pulp at 200°C. There was a significant decrease in cellulose composition at 225°C most likely due to initiation of cellulose degradation at this temperature, which was also evident from the release of glucose in the hydrolysate (Table 2). The lack of hydroxymethyl furfural peaks in the hydrolysate at 225°C suggest that glucose was relatively stable to thermal decomposition (Wang et al., 2019).

Despite substantial xylan degradation, recovery of xylose sugar was either low or even undetectable at 200 and 225°C, respectively (Tables 1, 2), which imply xylose degradation. This was evident from the generation of furfural, formic and acetic acids in the liquid hydrolysate at these temperatures. Acetic acid and secondary products such as furfural, carboxylic acids (formic, lactic and oxalic acids), aldehydes, dihydroxyacetone, and dark insoluble brown product from xylan degradation have been reported from treatments at subcritical temperatures (Jing and Lu, 2007; Pinkowska et al., 2011; Paksung and Matsumura, 2015). Furfural is produced mostly from dehydration of pentose sugars such as xylose and arabinose (Mandalika and Runge, 2012; Cai et al., 2014). Formic acid can be generated from intermediate products formed when furfural sequentially undergo hydration, dehydration, tautomerization and hydration reactions (Pinkowska et al., 2011). Insoluble dark sugar degradation products from caramelization of xylose sugar could have imparted the color on the pulp (Boonstra and Tjeerdsma, 2006; Jing and Lu, 2007). Pinkowska et al. speculated that diverse range of aldehydes, carboxylic acids and other sugar degradation products could be generated from xylan degradation at 180–300°C that could represent the unidentified peaks in the present study (Pinkowska et al., 2011). The mass balance in the present study could have been improved if these unidentified degradation products were quantified.

Among the degradation products, furfural has versatile applications as a solvent, platform chemical or ingredient in oil refinery, agriculture, pharmaceutical, and cosmetic industries (Mandalika and Runge, 2012; Peleteiro et al., 2016). It is produced by thermal hydrolysis of lignocellulosic biomass catalyzed by dilute acid as a sole product at low yields below 50% with high energy consumption for processing (Cai et al., 2014; Peleteiro et al., 2016). Improvements in the recovery process have increased the yields by up to 70%. Furfural was the major sugar degradation product generated from the hydrothermal treatment. There was substantial furfural yield [3.7 ± 0.2 and 4.1 ± 0.8 furfural (wt % original feedstock)] at 200 and 225°C treatments, respectively, that is equivalent to 19 and 21% xylan conversion (wt %). Hence, there is a potential to generate furfural as a value–added co–product with CNC through hydrothermal treatment–mediated acid hydrolysis process as a biorefinery strategy.

In addition to generation of furfural, hydrothermal treatment also increased the crystallinity index of the pulp (Table 1). The degree of crystallinity could have improved as a result of concentration of crystalline chains due to the significant degradation of xylan at temperatures ≥175°C. The other hypothesis is that hydrothermal treatment can also promote the reorientation of para–crystalline celluloses to form new compact crystals (Bhuiyan et al., 2000; Inagaki et al., 2010). Heat can rotate bond angles in the functional groups involved in hydrogen bonding between crystalline chains that facilitate higher level crystalline ordering (Agarwal et al., 2017). Isogai et al. reported an increase in crystallinity index of hydrothermally treated amorphous cellulose (Isogai et al., 1991). Inagaki et al. also observed that heat–treatment (140°C, up to 100 h) of raw wood conferred increase in the crystallite thickness (Inagaki et al., 2010). In addition, it is likely that xylan degradation promotes cellulose crystallization (Dwianto et al., 1996; Hult et al., 2001; Wan et al., 2010). The removal of the non–crystalline xylan interspersed between the cellulose chains can give more freedom of mobility for the para–crystalline celluloses to reorient and form compact crystals (Dwianto et al., 1996). The newly formed crystals conferred by hydrothermal treatment have been reported to be stable to subsequent soaking or washing (Bhuiyan and Hirai, 2005).

Hydrothermal treatment significantly increased CNC yield_1_, which suggests that heat treatment increased the CNC precursor composition of the feedstock going to the acid hydrolysis reactor (Table 3). CNC precursors can be concentrated due to xylan degradation or additionally from formation of new crystals. An improvement in CNC yield_1_ reduces the consumption of chemicals, water and, operational costs in upstream and downstream process as in the case of cellulase–mediated acid hydrolysis reported in our previous study (Beyene et al., 2017).

There was also significant improvement in CNC yield_2_ due to hydrothermal treatment even after factoring in the mass loss from the treatment that resulted from xylan degradation (Table 3). If the effect of hydrothermal treatment was only to concentrate the crystalline cellulose, then the CNC yield_2_ should have been constant. Therefore, the increase in CNC yield_2_ implies that heat treatment of wood pulp induced crystalline ordering that led to the formation new CNC precursors. It can be suggested that these newly formed crystals in the hydrothermally treated wood pulp significantly amplified the XRD intensities. In our previous study that explored cellulase treatment–mediated acid hydrolysis, XRD analysis was not sensitive to the changes in cellulase–treated pulp due to the concentration of CNC precursor even if this was implied from the improvements in CNC yield_1_ (Beyene et al., 2017, 2018). Agarwal et al. reported 8–19% CNC yield_2_ (lignin containing CNC) from hydrothermally treated (167–225°C) raw poplar wood, while the yield was negligible without the treatment (Agarwal et al., 2017). Therefore, partial crystallization of wood pulp cellulose by hydrothermal treatment improves the carbon use efficiency of the acid hydrolysis process. This strategy not only improves CNC productivity, which potentially generates more revenue, but also minimizes the sugar degradation products entering the acid stream to ease recovery. It was not surprising that CNC yields did not improve at 150°C as no transformation was evident from both compositional and crystallinity analysis of the hydrothermally treated wood pulp (Tables 1, 2). Taken together, this is the first report of a biorefinery strategy that introduces hydrothermal treatment prior to CNC isolation for the co–production of furfural and CNC with improved CNC yield.

The CNC isolated from hydrothermally treated pulp exhibited comparable characteristics relative to the control (Tables 4, 5). With regards to crystallinity, the newly formed crystals had equivalent level of crystalline ordering as the native crystals in wood pulp. Hence, fragmentation of these newly formed CNC precursors with the acid generated CNCs with similar degree of crystallinity relative to the control. This implies that crystallization only occurred on the para-crystalline domains as there was little or no room for significant re-orientation on the crystalline regions of the CNC precursors due to restriction from tight packaging. It is also possible that there was some level of crystallization that was not detected by XRD analysis. Agarwal et al. reported that crystallinity of CNC at 200°C was not substantially different from treatment at 170°C from XRD analysis, while 13.8 % improvement due to hydrothermal treatment was identified from Raman spectroscopy (Agarwal et al., 2018).

The hydrodynamic diameter of CNC was also not affected by hydrothermal treatment. The visually observed reduction in size of the square cut pulp to coarse and fine powder at 170 and 200°C, respectively (Figure 1), did not influence the CNC dimensions after acid hydrolysis. These results were also in agreement with CNCs from cellulase–treated pulp (Beyene et al., 2018). This implies that the permeability of CNC precursors to the acid under these conditions (64 wt % H_2_SO_4_, 2 h, 45°C) were conserved irrespective of the fiber size. The zeta potential, which is an indicator of surface charge from the esterified sulfonate group that confers colloid stability by electrostatic repulsion, did not change due to hydrothermal treatment until 225°C. This was further confirmed by the lower sulfur composition from elemental analysis of the CNC isolated from the treatment at this temperature. Degradation products bound to the surface of the hydrothermally treated pulp (Figure 1) may have prevented esterification of sulfonate groups. In addition, dehydration, decarboxylation and condensation reactions could have increased the carbon content and decreased the oxygen and hydrogen compositions (Boonstra and Tjeerdsma, 2006). The intense dark brown color could have been imparted on the CNC from caramelization reactions (Figure 3). Colored CNC could be undesirable for certain applications and caramelized products bound on the surface could likely affect the chemistry of the CNC and hence the reactivity with polymer matrices. Additional studies are required to identify purification strategies to generate colorless CNC from the hydrothermal treatment–mediated CNC isolation pathway.

## Conclusions

A novel biorefinery strategy that introduces hydrothermal treatment prior to acid hydrolysis was developed. This is the first demonstration of a pathway that co–produces furfural along with CNC from wood pulp feedstock. Hydrothermal treatment at 175–225°C degraded xylan and significant dehydration of the resulting xylose sugars to furfural was achieved with 20% conversion efficiency (wt % xylan) at 200 and 225°C. The addition of furfural into the product stream can open up new and growing markets for the pulp and paper industry as the molecule is a platform chemical and solvent for diverse products in various industries. In addition to furfural co–production, this is the first report of an improvement in CNC yield_1_ and yield_2_ due to crystallization of cellulose in a kraft pulp feedstock. These findings suggest that in addition to concentration of existing CNC precursor by degradation of xylan into furfural, hydrothermal treatment can also reorient para–crystalline cellulose chains in the wood pulp to form crystals that are equivalent to CNC precursors. Significant CNC yield improvement up to 4-folds was achieved, which will have significant impact not only in improving the CNC process cost but also production capacity, which makes integration of this pathway in CNC plants highly attractive and feasible.

## Data Availability Statement

The datasets generated for this study are available on request to the corresponding author.

## Author Contributions

DCB conceived the research idea. DCB and MC acquired funding and administered the project. DB conducted the experiments, carried out the data analysis and, prepared the first draft of the manuscript. All authors contributed to the experimental design, data interpretation, reviewed, and edited the manuscript.

## Conflict of Interest

The authors declare that the research was conducted in the absence of any commercial or financial relationships that could be construed as a potential conflict of interest.
